# Evaluation of the effectiveness of two hospitalization alternatives compared to standard psychiatric hospitalization

**DOI:** 10.1192/j.eurpsy.2023.2096

**Published:** 2023-07-19

**Authors:** A. Friedlander, D. Tzur Bitan

**Affiliations:** 1 Psychiatry, Sheba Tel-HaShomer Medical Centre, Ramat Gan; 2 Behavioral Sciences, Ariel University, Ariel; 3 Psychiatry, Shalvata Mental Health Center, Hod Hasharon, Israel

## Abstract

**Introduction:**

In recent years there has been an ongoing search for alternatives to psychiatric hospitalizations, which might overcome the barriers of social stigma and institutionalization. Nonetheless, there is a paucity of empirical studies which address the effectiveness of these potential alternatives as compared to the traditional medical model.

**Objectives:**

The purpose of the current study is to compare the effectiveness of psychiatric hospitalization with two alternatives: Soteria homes, which emphasize the cultivation of empathetic and non-intrusive relations, and technologically assisted home hospitalization, which places the emphasis on the provision of psychiatric care in a manner which resembles the medical model, but in close proximity to the patient’s family.

**Methods:**

Subjects and staff completed self-report measures of their symptomatic distress, social functioning, interpersonal relations, quality of life, self-stigma, therapeutic alliance, global functioning (GAF) and positive and negative symptoms of schizophrenia (PANSS). Measurements were completed at baseline, at discharge, and at three months, half a year, a year and a year and a half after discharge.

**Results:**

Overall, the study included 214 subjects, which were non-randomly allocated to one of the three acute care modules: psychiatric hospitalization (N=66), Soteria homes (N=94) and technologically assisted home hospitalization (N=54). The average age in the total sample was 36 years (SD=14.2) and 49.1% of them were women. The majority of patients (47.7%) were diagnosed with a psychotic or bipolar diagnosis, followed by mood disorders (29.4%), PTSD or a personality disorders (19.6%) and others (3.3%). About 68.5% earned less than the average, 10.2% earned an average salary and 21.4% earned above the average.

**Conclusions:**

Full description of the results elaborating on the differences between treatment modules across the three acute care settings will be presented and discussed. The results of the current study can provide significant insights about the effectiveness of psychiatric hospitalization alternatives for acute mental states and can influence decision-making processes and policy trends worldwide.

**Disclosure of Interest:**

None Declared

